# A Case of Focal Liver Necrosis Following Whipple’s Procedure: Presentation, Workup, and Management

**DOI:** 10.7759/cureus.98722

**Published:** 2025-12-08

**Authors:** Isuri S Rathnayake, Kangaiyanan Sivarajah, Mahanama Gunasekara, Sasindu S De Silva

**Affiliations:** 1 Surgery, Lady Ridgeway Hospital for Children, Colombo, LKA; 2 Surgery, National Hospital of Sri Lanka, Colombo, LKA

**Keywords:** aberrant gastric artery, elevated liver enzymes, liver abscess, liver necrosis, mesenteric angiogram, whipple’s procedure

## Abstract

Liver necrosis, liver abscess, and mesenteric ischemia are uncommon but serious complications of Whipple’s procedure. Hepatic arterial tree assessment before surgery and meticulous surgical technique may prevent liver injury. We present a case of focal liver necrosis of the left lobe following Whipple’s procedure due to hepatic arterial cut off, with minimal clinical effects due to an aberrant arterial connection of the left gastric artery to the left hepatic artery. A 53-year-old female underwent Whipple’s procedure for a neuroendocrine tumor of the head of the pancreas. Forty-eight hours later, liver enzyme levels were high, and direct bilirubin levels were elevated. The duplex ultrasound scan showed a normal portal venous flow. Hepatic arterial flow was not visualized. She was managed with a liver failure regimen and recovered within a week. The patient was re-admitted with a purulent discharge from the surgical incision on postoperative day 30, which tested negative for amylase and microbial growth. CT of the abdomen showed a necrotic segment of the left lobe of the liver with an abscess tracking into the skin. However, the liver biochemistry was normal. Mesenteric angiography showed an abrupt cut-off of the hepatic artery at the common hepatic artery origin. However, right and left hepatic artery flow was intact distally to the bifurcation, due to a communicating collateral artery from the left gastric artery to the left hepatic artery. She underwent a laparotomy. Necrotic material was washed out, and a drain was inserted. Outpatient care with monthly ultrasound scans was performed. Drain output gradually reduced. The patient resumed her daily activities and preoperative body weight. A follow-up CT at eight months showed minimal hepatic collection. Subsequently, the drain was removed. Hepatic arterial flow disruption can present as transient liver failure, focal liver necrosis, and abscess formation. Liver necrosis and abscess can be managed by initial medical treatment and percutaneous surgical drainage. Collateral arterial pathways to the hepatic artery may minimize the extent of liver injury.

## Introduction

Despite technological advances and developments, pancreateco-duodenectomy (Whipple’s procedure) is still considered a challenging surgery due to its operative morbidity and mortality. Hospital mortality was approximately 25% during the early 1960s, which decreased to nearly 5% by the late 1990s [1]. Morbidity ranges from 30% to 60%, which includes pancreatic fistulae, anastomotic leaks, bile leaks, delayed gastric emptying, wound-related complications, pulmonary complications, and ischemic complications [2,3].

Ischemic complications following Whipple’s are rare but can be devastating, and include mainly hepatic, bile duct, or bowel ischemia. Main reasons are intraoperative injury to the main blood vessels or aberrant vessels, pre-existing arterial diseases, or postoperative embolism. Liver ischemia and/or focal necrosis after Whipple’s procedure are uncommon complications. Transient liver ischemia due to intraoperative hypoperfusion or cardiac events mostly recovers during the postoperative period.

However, pre-existing vascular anomalies including stenosis of the superior mesenteric artery (SMA) or hepatic artery due to atherosclerosis, vascular anomalies such as replaced right or left hepatic artery (an anatomical variation where the hepatic artery originates from the SMA instead of the normal common hepatic artery, a variation that occurs in about 9-15% of people) which were undiagnosed at the time of surgery, or intraoperative damage of hepatic artery or portal vein can give rise to persistent ischemic liver complications, including segmental necrosis and acute liver failure [2].

Management of ischemic liver complications depends on several factors, including the patient’s clinical condition, cause of the ischemia, and available expert facilities for revascularization procedures. Revascularization in the early stages can prevent extensive ischemic injury; however, radiological or surgical drainage of focal necrotic material can also be considered in less extensive necrosis.

We present this unique case with segmental liver necrosis following Whipple’s procedure, discuss the diagnostic and management challenges, and emphasize the role of collateral circulation.

## Case presentation

Preoperative presentation

A 53-year-old female with type 2 diabetes mellitus and hypertension presented with a significant loss of appetite and weight loss. She had no history of jaundice or serotonin syndrome. The initial body mass index was 16.8 kg/m². There was an epigastric mass on palpation.

**Figure 1:**
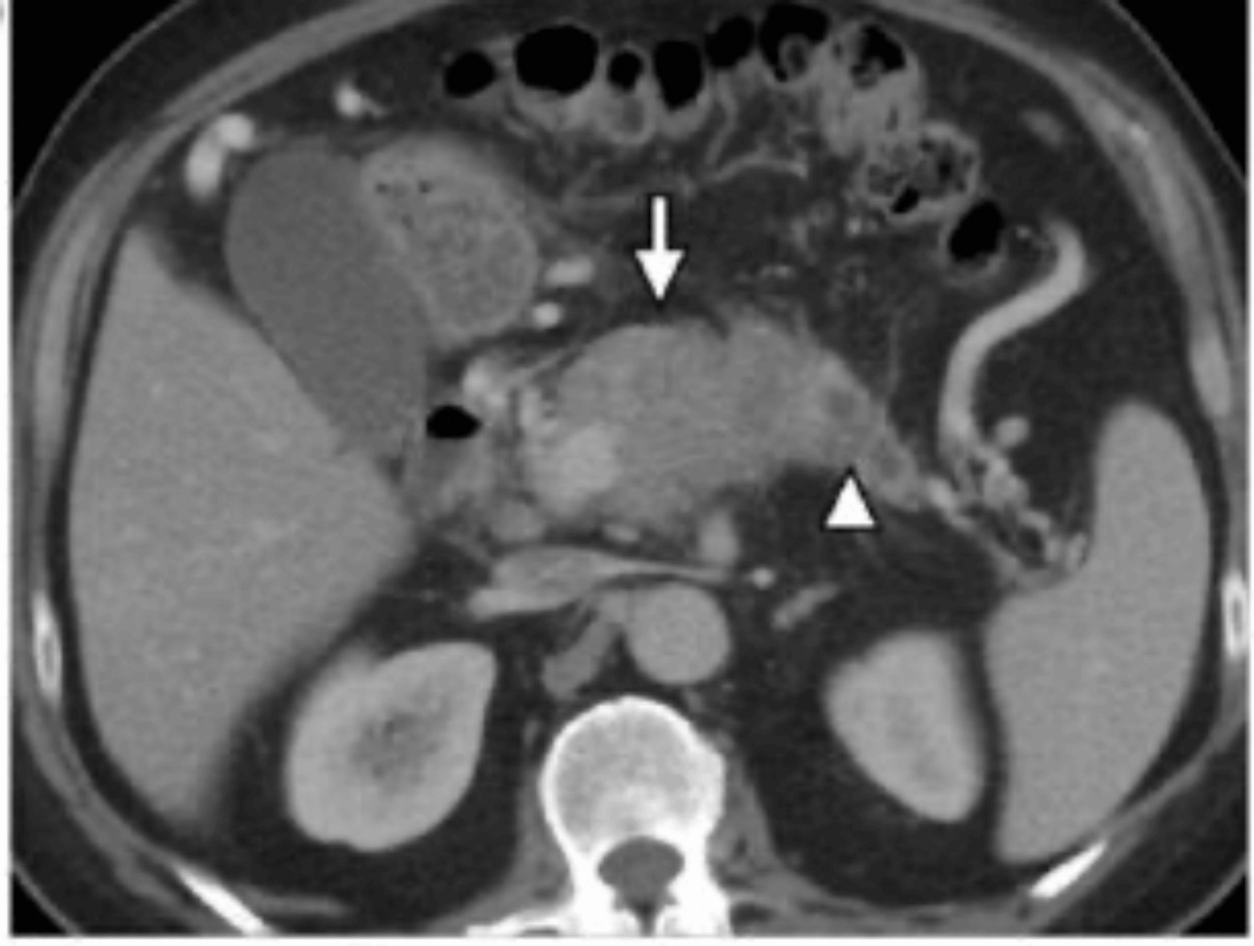
Preoperative image of the pancreatic neuroendocrine tumor. The white arrow shows the tumor of the head.

Preoperative hemoglobin and liver functions were normal. Tumor markers of carbohydrate antigen 19-9, chromogranin A, serum gastrin, and 24-hour urine 5-hydroxy3-indolacetic acid levels were normal. Contrast-enhanced CT (CECT) of the abdomen showed a well-defined lesion in the head of the pancreas measuring 4 cm × 4 cm × 3.6 cm, as shown in Figure 1. Major arteries and veins were devoid of tumor encasement. Endoscopic ultrasound and fine needle aspirate cytology showed a neuroendocrine tumor.

Operative details

The patient underwent standard Whipple’s surgery for five hours. All major vascular structures were devoid of tumor. Intraoperative hypotension (mean arterial pressure: 53 mmHg) was detected and managed with inotropic support.

Immediate postoperative course

**Figure 2:**
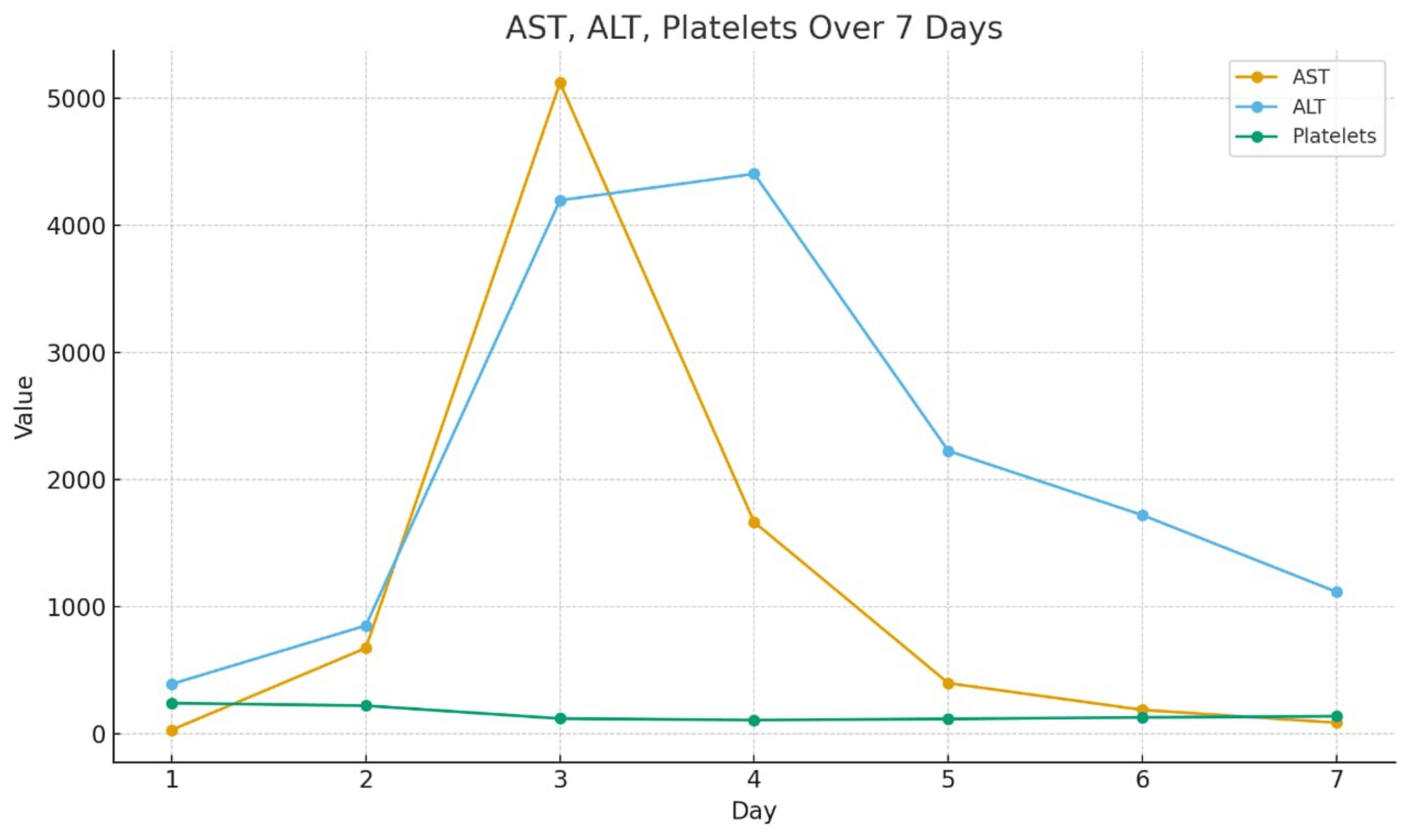
Trend of key biochemical parameters. AST = aspartate aminotransferase; ALT = alanine aminotransferase

On intensive care unit (ICU) day two, aspartate aminotransferase (AST) and alanine aminotransferase (ALT) levels started to rise above 5,000 U/L, as shown in Table 1 and Figure 2. The patient showed reversal of sleep pattern, indicating grade 1 hepatic encephalopathy, according to the West Haven criteria for encephalopathy. Both features indicated acute liver failure. The patient was managed with intravenous N-acetylcysteine infusion, oral rifaximin, lactulose, and vitamin K.

**Table 1:** Pattern of biochemical markers on postoperative days. AST = aspartate aminotransferase; ALT = alanine aminotransferase; PLT = platelet; CRP = C-reactive protein; INR = international normalized ratio; ALP = alkaline phosphatase

Marker	Preoperative value	Reference range	Day 1	Day 2	Day 3	Day 4	Day 5	Day 6	Day 7	Day 8	Day 16
AST (IU/L)	19	5–40	32	676	5,120	1,666	400	190	89	91	25
ALT (IU/L)	20	7–56	393	853	4,196	4,040	2,226	1,721	1,117	833	20
Total bilirubin/Direct bilirubin (mg/dL)	1/0.6	0.2–1.2 /0–0.4	0.9/0.4	2.2/0.7	1./0.6	1.6/0.6	-	1.2/0.6	1/0.6	-	1/0.6
INR	1.1	-	1.4	1.6	2.2	2.0	1.42	-	1.5	-	1.1
ALP (U/L)	100	<34	-	-	208	-	-	-	169	-	98
PLT (/mL)	250	150–450/mL	243	223	122	110	119	131	140		240
CRP (mg/dL)	0.5	mg/dL	-	-	-	-	129	-	-	-	5

Ultrasound of the abdomen with Doppler study was performed on postoperative day three, which showed normal liver echogenicity, with normal diameter and velocity of flow (18 cm/second) in the portal vein, without evidence of thrombosis of the portal vein and minimal free fluid in the abdomen. The operator had not commented about the flow in the hepatic artery.

Despite the transient liver derangement, vital parameters were maintained within the expected goals. By the sixth to seventh postoperative day, the patient’s liver functions improved (ALT: 60%, AST: 90%), along with the clinical symptoms of liver failure, as shown in Table 1. She was started on clear fluids via a nasogastric tube on postoperative day three. The time to a proper meal was seven days. She was given total parenteral nutrition from day three onwards.

She was discharged from the ward on postoperative day 16 with a healthy abdominal wound. Histology and immunohistochemistry showed positivity for synaptophysin and chromogranin A, with a Ki index of 4% suggestive of a grade 2A pancreatic neuroendocrine tumor with R0 resection.

Late presentation and management

The patient was reviewed in the clinic, and around 50 days later, was found to have a purulent discharge from her laparotomy scar. The patient was afebrile, and hemodynamic parameters were stable except for mild dehydration. Abdominal examination revealed a purulent discharge from the proximal edge of the midline laparotomy incision but no generalized peritonitis. Pus was sent for cultures and amylase levels. However, the pus was negative for amylase but positive for coliform. As there was a perioperative liver injury, we decided to evaluate her, suspecting a liver abscess.

**Figure 3:**
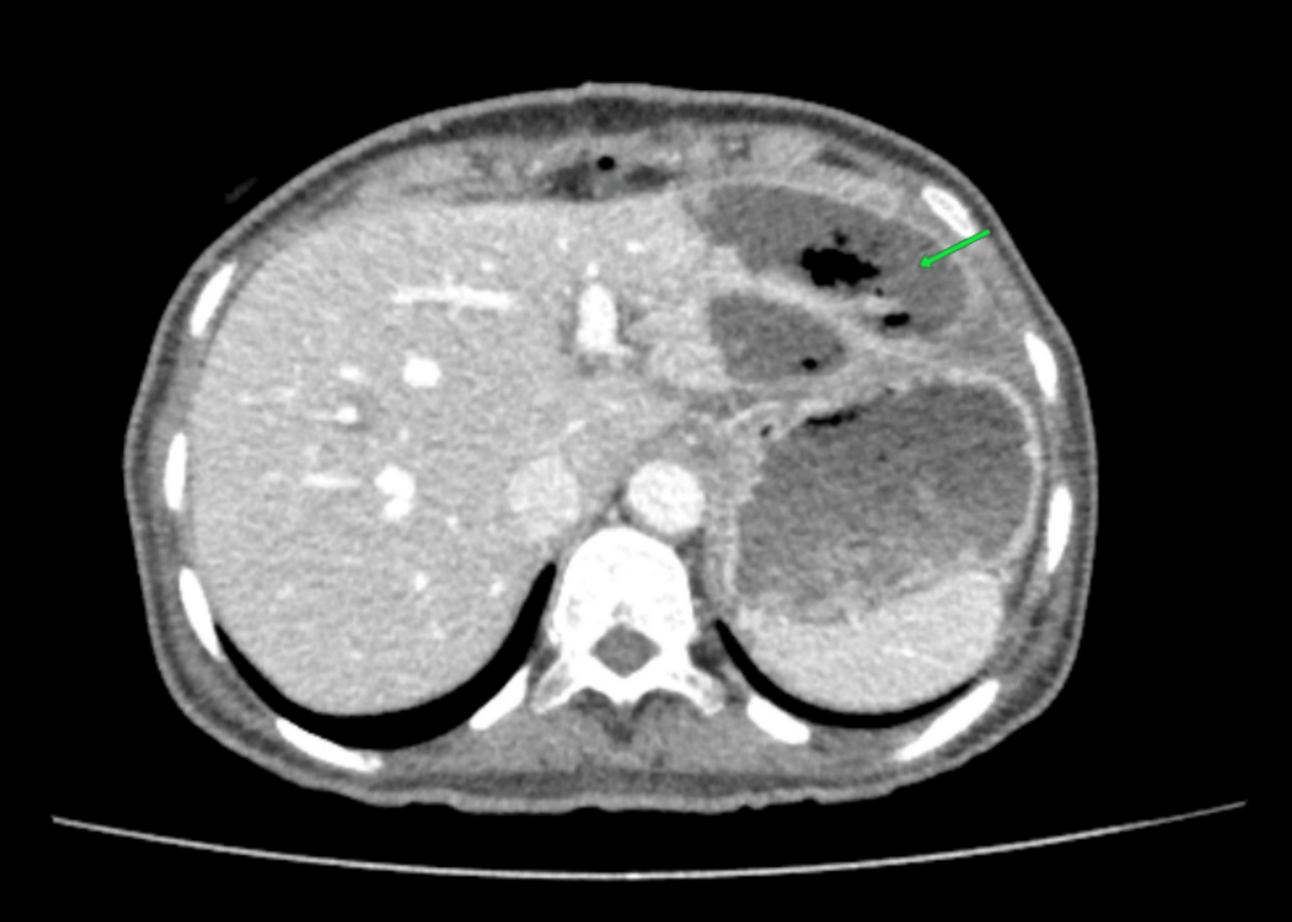
Left liver lobe necrosis in segments 2 and 3. The green arrow shows the necrotic area measuring 3 cm × 5 cm on the left lobe.

**Figure 4:**
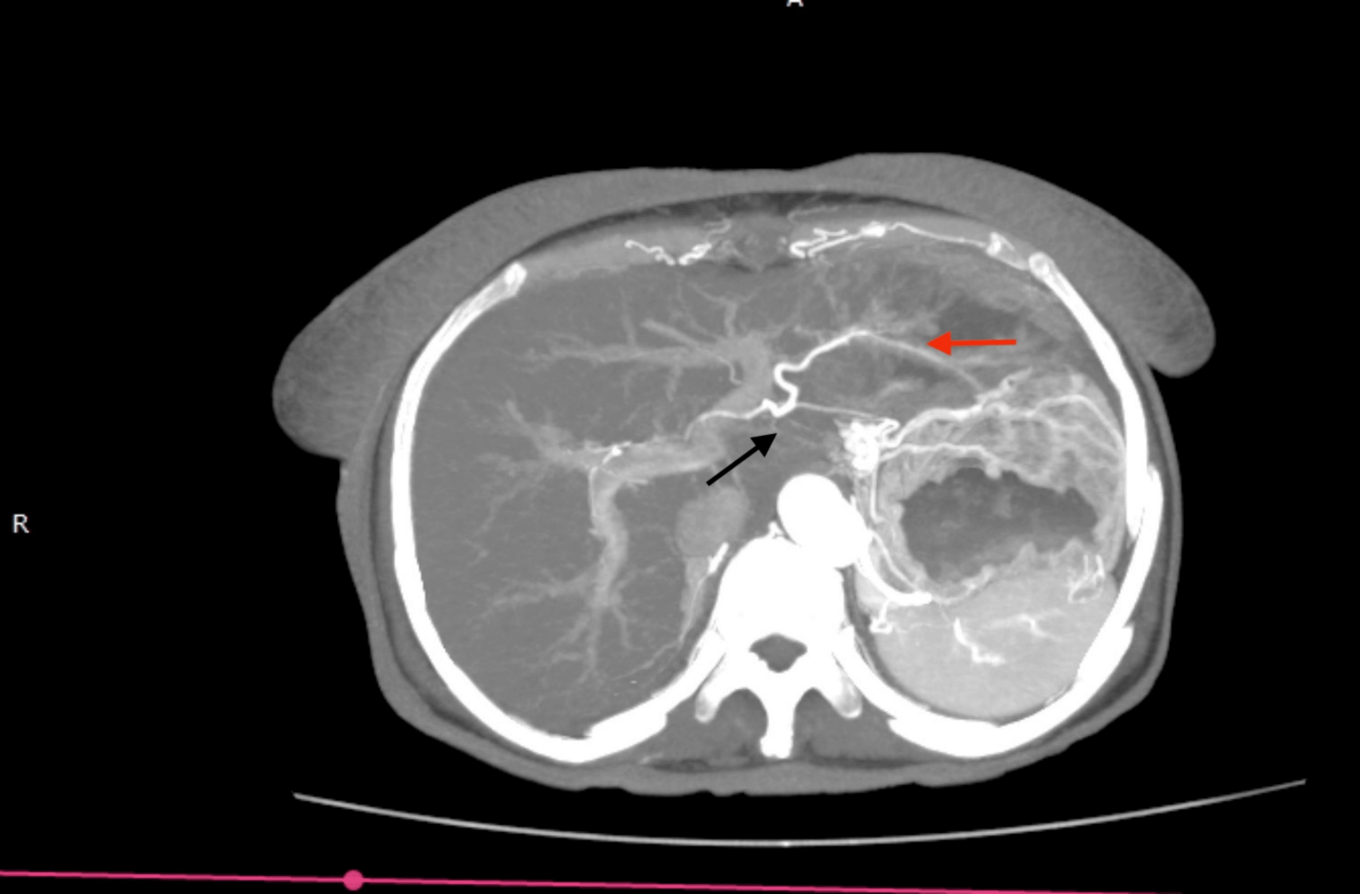
Mesenteric angiogram (arterial phase) showing the abrupt cut-off of the common hepatic artery and aberrant left gastric artery connecting the left hepatic artery. The black arrow shows the common hepatic artery (none opacified) with right and left hepatic artery confluence. The red arrow shows the aberrant left gastric artery flow into the left hepatic artery.

CECT of the abdomen with mesenteric angiogram was performed and showed an abrupt cutoff of flow in the common hepatic artery 2 cm from the celiac trunk, with necrosis in the segments 2 and 3 of the liver with abscess formation (Figure 3). However, the confluence of right and left hepatic arteries showed contrast filling from the branch of the left gastric artery (Figure 4). The left branch of the portal venous flow was diminished. The main portal vein and the right portal vein demonstrated normal flow.

Management of focal liver necrosis

Initially, an ultrasound-guided drain was inserted, following which the patient underwent a laparotomy and insertion of a drain. She was hospitalized for two weeks and then managed as an outpatient with the drain. Abdominal ultrasound scans were performed every two months to assess the collection and other complications. She was given a nutritionist plan during each visit.

Follow-up CECT and angiogram performed at six months postoperatively showed a thick (40 HU) residual collection measuring 4 × 2 × 2 cm in the most peripheral part of the left liver lobe. No evidence of anastomosis leak or tumor recurrence was seen. The celiac axis was normally opacified, but the common hepatic artery showed severe stenosis with minimal flow in the right hepatic artery and no flow in the left hepatic artery. The patient was educated regarding the condition, and she maintained good compliance with the treatment and the nutritionist’s plan.

## Discussion

Incidence of liver ischemia after pancreatic-duodenectomy is relatively uncommon, ranging from 0.5% to 2.7% [1,4]. Ischemic complications, including mesenteric ischemia or liver necrosis, were found to be the leading cause of death in a series of 545 patients who underwent pancreatico-duodenectomies [1].

Operative injury to the common hepatic artery, portal vein or both, accidental ligation of a replaced right or left hepatic artery which was not identified before surgery, pre-existing vascular anomaly such as stenosis of the celiac trunk or SMA, thromboembolism due to operative handling of the vessels and perioperative hypotension due to bleeding, epidural anesthesia, or cardiac complications can be listed as the causes for postoperative hepatic perfusion defects [1,5,6]. The incidence of hepatic artery injury ranges from 0.5% to 2.7% [7]. Mechanisms of trauma include inadvertent transection and arterial thrombosis or pseudoaneurysm secondary to traumatic dissection. In this index case, operative injury to the common hepatic artery or postoperative thrombosis and subsequent stenosis of the common hepatic artery is most likely.

Reduction of hepatic perfusion can be suspected at the time of surgery by inspection for macroscopically pale liver, looking for pulsations of the hepatic artery pedicle, and assessing reduced bile flow from the stump [7]. However, this can be surgeon-dependent. Intraoperative duplex ultrasound can be performed to diagnose hepatic artery injury [7].

Clinical presentation of hepatic ischemia can be variable; some manifest in the first postoperative day or may be delayed up to several months [7]. Clinical manifestations could be simple postoperative liver enzyme elevation, acute liver failure, sepsis with abdominal collection, fulminant liver failure, segmental necrosis and or abscess formation, biliary leaks, and biliary stenosis, among others [7]. If both the portal vein and hepatic artery are injured, mortality will be 100% [5]. This case had an atypical presentation where the initial liver injury was limited due to an aberrant artery supplying the right lobe of the liver, leading to necrosis and abscess formation segmentally and presenting late as a purulent discharge from the surgical site.

Transient elevation of serum transaminase levels on the first postoperative day, i.e., ALT and AST, could be a normal phenomenon in pancreatic-duodenectomy due to the operative handling of the liver, blood loss, and effects of epidural-induced hypotension, where the levels usually elevate to about 100 U/L [5]. Acute liver failure refers to the development of acute severe liver injury with encephalopathy and impaired synthetic function (international normalized ratio: >1.5) in a patient without cirrhosis or preexisting liver disease, elevated aminotransferase and bilirubin levels (>10 mg/dL), and low platelet count [8]. Transaminase levels of more than 1,000 U/L have been correlated with a positive duplex finding in hepatic perfusion defects [9]. The pattern of elevation of liver enzyme levels (Table 1) and their recovery in this case is consistent with similar case scenarios reported in the literature [5].

Clinical and biochemical suspicion of liver injury should be combined with radiological investigations to identify possible etiological factors and associated other postoperative complications. Duplex ultrasound can be used to detect portal vein thrombosis, hepatic arterial stenosis, or thrombosis, as it is easy to perform in the ICU settings. It has shown a sensitivity of 87%-100% and a specificity of 96-97% in detecting hepatic arterial defects and a sensitivity of 77% and a specificity of 97% for portal vein thrombosis. Drawbacks are that it is operator-dependent and, in early postoperative collections, tissue edema can hinder the quality of the images [10,11]. A negative duplex finding for hepatic arterial flow in this case could have been due to operator dependency or the quality of images that were available in the low-resource setting.

CT mesenteric angiogram is more sensitive than duplex studies in the identification of arterial thrombosis or stenosis. Furthermore, it has the added advantages of detecting other complications related to surgery, such as anastomotic leaks, liver necrosis, or abscess, suggestive of ischemic complications [10-14]. Liver abscess can be challenging to differentiate from the infarction, but the pattern of elevation of transaminase levels combined with the CT findings will be more definitive [15]. In this study, the CT angiogram was performed after 30 days of initial elevation of AST and ALT. We would recommend a CECT of the abdomen with an angiogram at the initial stage for a similar case with an initial liver derangement.

The liver has a dual blood supply from the portal venous system (80%) and hepatic arteries (20%). The portal vein provides most of the nutrients and 50% of the oxygen demand. It has also been demonstrated that many collateral arterial pathways to the liver exist, mainly the gastroduodenal and right gastric arteries [16-18]. Thus, the liver is considered to have good ischemic tolerance. In this case, there was an abrupt cutoff of the common hepatic artery. However, a collateral pathway connecting the left hepatic artery and the right gastric artery was demonstrated (Figure 4). This fact and the combination of the preserved portal venous flow could have been the reason that only left-sided segmental necrosis occurred instead of global necrosis.

There are no standard recommendations for managing hepatic infarction following hepatobiliary surgery. It has to be tailored to each clinical presentation, depending on various factors such as the clinical condition of the patient, cause of the hepatic infarction, available expertise and facilities, and the overall prognosis of the patient.

In a series of 11 patients with postoperative hepatic infarction, caused by an inadvertent hepatic artery injury during surgery, by accidental ligation of the right hepatic artery, or long-standing clamping, three patients had undergone intravenous administration of prostaglandin E1 (PGE1) for fulminant liver failure, which increased hepatic blood flow and led to better clinical outcomes for reperfusion injury [19]. Other options for management included conservative management without surgical interventions and plasma exchange and dialysis for organ support.

When the hepatic artery injury is the culprit for hepatic ischemic complications, a more comprehensive management strategy has been suggested by Landen et al. [7] in their systematic review, which concluded that immediate arterial repair would prevent major life-threatening complications, such as liver necrosis, acute liver failure, biliary fistula, and strictures, and to consider immediate revascularization procedures when the liver enzyme levels are above 2,000 IU.

Options of immediate revascularization include saphenous vein interposition, inferior mesenteric vein interposition, direct anastomosis, and gastroduodenal artery transposition. Delayed management options include hepatic artery stenting, delayed grafts, or redo anastomosis for biliary strictures. However, this decision depends on the available technical resources, presence of collateral arterial supply, biliary obstruction and sepsis, site of the arterial injury, and the quality of the portal flow. Surgical thrombectomy was also performed in a few cases where a hepatic artery or graft thrombus was suspected. In this case, common hepatic artery revascularization was not considered, as this was a delayed presentation and there was an existing liver abscess and local sepsis at the time of diagnosis.

Once hepatic necrosis and abscess on imaging is established, management options are mainly either surgical debridement or serial surveillance with or without percutaneous or surgical draining of the necrotic collections [20] with appropriate antimicrobial treatment and nutritional support, for which this patient was subjected to and subsequently improved.

## Conclusions

Liver ischemia following a pancreateco-duodenectomy is relatively uncommon. It can be due to arterial or venous injury or perioperative hypotension. Hepatic arterial injury can present as transient liver failure, focal liver necrosis, and abscess formation. Diagnosis is a combination of clinical presentation, elevated transaminases, and positive imaging. CECT with angiogram is more useful than duplex ultrasound scans in identifying liver ischemia. Existing collateral pathways to the hepatic arteries from the gastric arteries may minimize the extent of the liver injury. A preoperative detailed angiogram can be recommended to identify any preexisting vascular anomalies of the SMA and common hepatic artery before the pancreateco-duodenectomy procedure. An early CT angiogram will be more helpful in identifying hepatic arterial injury during the perioperative period. Arterial revascularization or repair can be attempted depending on the clinical parameters, timing of diagnosis, local sepsis, and available resources. Liver abscess with fistulation can present as a delayed purulent wound discharge. Once a hepatic abscess is diagnosed, it can be managed by percutaneous drainage or surgical debridement and follow-up.
